# One Health surveillance of multidrug-resistant *Escherichia coli* in broilers and market environments in Aceh, Indonesia: A triangulated sampling approach

**DOI:** 10.14202/vetworld.2025.3149-3161

**Published:** 2025-10-26

**Authors:** Teuku Reza Ferasyi, Mahdi Abrar, Ismail Ismail, Wahyu Eka Sari, Azhari Azhari, Mustafa Sabri, Faisal Jamin, Erwin Erwin, Siti Rani Ayuti, Mirni Lamid

**Affiliations:** 1 Laboratory of Veterinary Public Health, Faculty of Veterinary Medicine, Universitas Syiah Kuala, Banda Aceh, Indonesia; 2 Center for Tropical Veterinary Studies-One Health Collaboration Center, Universitas Syiah Kuala, Banda Aceh, Indonesia; 3 Laboratory of Microbiology, Faculty of Veterinary Medicine, Universitas Syiah Kuala, Banda Aceh, Indonesia; 4 Laboratory of Anatomy, Faculty of Veterinary Medicine, Universitas Syiah Kuala, Banda Aceh, Indonesia; 5 Laboratory of Clinics, Faculty of Veterinary Medicine, Universitas Syiah Kuala, Banda Aceh, Indonesia; 6 Doctoral Program of Veterinary Science, Faculty of Veterinary Medicine, Airlangga University, Surabaya, Indonesia; 7 Division of Animal Husbandry, Faculty of Veterinary Medicine, Airlangga University, Surabaya, Indonesia

**Keywords:** antimicrobial resistance, broiler chicken, *Escherichia coli*, Indonesia, One Health, traditional markets

## Abstract

**Background and Aim:**

Multidrug-resistant (MDR) *Escherichia coli* in poultry poses a critical threat to food safety and public health. While studies have assessed resistance at the farm level, limited attention has been given to informal market environments that connect animals, humans, and surfaces. This study applied a One Health triangulation sampling approach to investigate the occurrence and antimicrobial resistance (AMR) profiles of *E. coli* isolated from broiler chickens and associated environments in traditional markets in Aceh Besar District and Banda Aceh City, Indonesia.

**Materials and Methods:**

A cross-sectional study was conducted in three traditional markets (Lambaro, Al-Mahirah, and Seutui). A total of 174 samples were collected, comprising fecal swabs (n = 54), chicken meat swabs (n = 54), poultry sellers’ hand swabs (n = 48), and chicken display table swabs (n = 18). Isolation of *E. coli* was performed using culture and biochemical confirmation. Antimicrobial susceptibility was tested using the Kirby–Bauer disk diffusion method against 11 antibiotics commonly used in veterinary and human medicine.

**Results:**

Overall, *E. coli* was isolated from 31.03% (54/174) of samples. Contamination was highest in fecal samples (13.79%), followed by chicken meat (8.62%), sellers’ hands (4.60%), and display tables (4.02%). Market-level prevalence was highest at Al-Mahirah (13.2%), followed by Lambaro (11.49%) and Seutui (6.32%). All isolates exhibited MDR phenotypes. Distinct variation in resistance profiles was observed between sample types in a range of 12.5%-100%. Percentage of resistance of isolates from all sample types were uniformly high to ampicillin (100%). Isolates from fecal and display table were 100% resistant to erythromycin and kanamycin, as well as to streptomycin. The variation of resistance profiles from each sample types were also observed between markets.

**Conclusion:**

Traditional poultry markets represent critical hotspots for AMR dissemination at the human–animal–environment interface. Findings highlight the combined influence of unregulated antibiotic use in poultry production and inadequate hygiene practices on sustaining MDR *E. coli*. The triangulated One Health design demonstrates the added value of integrating animal, human, and environmental sampling for AMR surveillance. Strengthening antimicrobial stewardship, upgrading market hygiene infrastructure, and expanding integrated surveillance into national AMR monitoring frameworks are essential steps to mitigate public health risks.

## INTRODUCTION

The rising prevalence of antimicrobial resistance (AMR) in farm animals has become a major global concern [1, 2]. In Southeast Asia, including Indonesia, increasing antimicrobial use and the subsequent emergence of resistant strains within the livestock sector have been widely reported [3]. This trend is largely attributed to the excessive and often inappropriate application of antibiotics in poultry farming, where they are used as growth promoters or for disease prevention, thereby accelerating the development of multidrug-resistant (MDR) bacteria [2–5]. The occurrence of MDR in *Escherichia coli*, a ubiquitous bacterium in poultry and other livestock, ranges from harmless commensal strains to pathogenic variants [6, 7]. In Indonesia, most investigations into AMR have focused on farm-level detection of resistance profiles in chicken samples [8, 9]. By contrast, studies examining AMR in post-farmgate environments, such as traditional markets and slaughterhouses, remain scarce, particularly in developing countries. Traditional markets are of particular concern because they provide conditions that allow resistant bacteria to persist, disseminate, and directly expose consumers, yet these hotspots remain underexplored. Previous studies in Indonesia have highlighted this risk. Rizal *et al*. [7] and Suswati *et al*. [10] reported a high prevalence of *E. coli* resistant to multiple antibiotics in broiler meat and cloacal swabs from traditional markets in Cibinong (West Java) and Jember (East Java). Similarly, Wibawati *et al*. [11] identified widespread resistance to several antibiotics in *E. coli* isolates from broiler meat collected in East Java slaughterhouses, with 59.3% of isolates exhibiting MDR phenotypes. Collectively, these findings underscore the mounting public health threat posed by AMR, which compromises food safety, facilitates zoonotic transmission, and diminishes the effectiveness of critical antibiotic therapies [1, 12, 13].

Despite the increasing number of literature on AMR in livestock production, most studies in Indonesia and other Southeast Asian countries have concentrated on farm-level surveillance, focusing on cloacal swabs, fecal samples, or broiler meat collected directly from farms [8, 9]. While these studies provide valuable insights into resistance patterns at the production stage, they often overlook post-farmgate environments, where poultry products are handled, processed, and sold. Traditional markets, in particular, represent critical nodes of exposure, as they are characterized by limited hygiene infrastructure, high human–animal interaction, and close contact between raw meat and consumers. These conditions create favorable environments for the persistence and spread of MDR bacteria. However, evidence from these markets remains extremely limited. Previous studies by Rizal *et al*. [7], Suswati *et al*. [10], and Wibawati *et al*. [11] in Indonesia have documented MDR *E. coli* in broiler meat and cloacal swabs from traditional markets in West and East Java, but comprehensive investigations that capture contamination pathways beyond meat, such as sellers’ hands and display surfaces, are lacking. Furthermore, few studies globally have applied a triangulated One Health approach that simultaneously integrates animal, human, and environmental sampling within the same market ecosystem. This methodological gap leaves critical uncertainties regarding the full spectrum of AMR transmission routes in informal poultry markets and their contribution to foodborne public health risks.

To address these gaps, this study applied a One Health triangulation sampling framework to investigate the occurrence and AMR patterns of *E. coli* isolated from broiler chickens and associated environments in traditional markets in Aceh Besar District and Banda Aceh City, Indonesia. Specifically, the study aimed to (i) determine the prevalence of *E. coli* across multiple transmission pathways, including fecal samples, chicken meat, poultry sellers’ hands, and display surfaces; (ii) characterize the resistance profiles of these isolates against 11 commonly used antibiotics in both veterinary and human medicine; and (iii) compare resistance patterns across different market locations and sample sources. By integrating animal, human, and environmental compartments within the same market setting, this study provides a more holistic understanding of AMR ecology in traditional markets. The findings are expected to inform risk-based interventions, support the development of integrated surveillance strategies, and guide antimicrobial stewardship policies within Indonesia and comparable settings where informal markets dominate food systems.

## MATERIALS AND METHODS

### Ethical approval and biosafety considerations

This study was approved by the Research Ethics Committee of the Faculty of Veterinary Medicine, Universitas Syiah Kuala (Approval No: [387/KEPH/V/2021], Date: May 10, 2021). All sample collection procedures complied with institutional guidelines for the ethical handling of animal and environmental samples. Ethical clearance for human participants was obtained through verbal consent.

The laboratory procedures involving the isolation and handling of MDR *E. coli* were conducted in accordance with standard biosafety protocols at the Laboratory of Microbiology, Faculty of Veterinary Medicine, Universitas Syiah Kuala. The procedures consisted of using personal protective equipment (PPE), including laboratory coats, gloves, and masks, restricting access to the working area, and adhering to aseptic techniques during culture handling. The biological waste and used culture media were decontaminated by autoclaving at 121°C for 15 min before disposal.

### Study period and location

The study was conducted from June to August 2021 in Aceh Besar District and Banda Aceh City, Indonesia. Banda Aceh, the provincial capital, has a population of approximately 270,000, while the Aceh Besar District has more than 400,000 inhabitants. Banda Aceh and Aceh Besar have a humid tropical climate, characterized by high annual rainfall (2,000–3,000 mm) and average temperatures ranging from 26°C to 32°C. Both areas have a high demand for poultry products, which also serve as the primary distribution points for broiler meat.

Traditional poultry markets in Banda Aceh and Aceh Besar are located in public market complexes. The poultry trade is conducted in semi-permanent stalls, often with limited hygiene infrastructure. Markets usually operate from early morning to midday, with peak sales before noon. More than 80% of the broiler chickens brought to these traditional markets are sourced from smallholder farms in other districts. Some of them were supplied from farms in the neighboring province of North Sumatra.

### Study design

**Figure 1:**
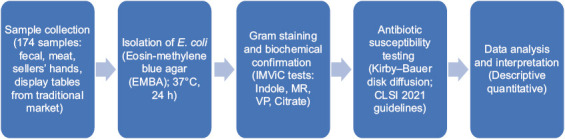
Flowchart of materials and methods.

This study was designed as a preliminary cross-sectional study to investigate the occurrence and AMR patterns of *E. coli* across the poultry supply chain. A total of 174 samples were collected from three traditional poultry markets. Figure 1 presents a schematic flowchart of the materials and methods applied in this study, covering the sequential steps from sample collection, bacterial isolation, and biochemical confirmation to antibiotic susceptibility testing.

### Sample collection

The target sample size was determined based on logistical feasibility, considering resource availability, time constraints, and the need to represent multiple potential transmission pathways (animal, human, and environmental). Therefore, this study should be considered as a preliminary surveillance study.

Samples were collected from three traditional markets in Aceh Besar District and Banda Aceh City. A total of 58 samples from each market were collected using the convenience sampling method, which comprised 18 fecal samples, 18 chicken meat swab samples, 16 swab samples from sellers’ hands, and 6 swab samples from chicken display tables. The decision to use 58 samples per market was based on a balance between logistical feasibility, resource availability, and the need for adequate representation of the animal-human-environment interface in each market.

Sampling was conducted during the morning market operating hours (7:00–10:00 am), when poultry transactions were most active. Collections were conducted on three separate market days, both on workdays and weekends, per location within the same month, to reduce temporal bias. Samples were randomly selected from available sellers within each market to avoid systematic bias in sample collection.

All swabs were immediately placed into sterile tubes containing Buffered Peptone Water (Oxoid, UK) as a transport medium, which supports the recovery of stressed enteric bacteria. All collected samples were transported to the laboratory in an ice box container and maintained at a temperature of 4°C–8°C during transport to prevent bacterial overgrowth or degradation. The collected samples were analyzed for bacteriological culture by the Laboratory of Microbiology, Faculty of Veterinary Medicine, Universitas Syiah Kuala. Upon arrival, the samples were either processed immediately or stored at 4°C and processed within 24 h to minimize changes in microbial viability and resistance profiles.

The sample types were selected to reflect the One Health transmission pathways: Fecal and chicken meat samples represent potential animal-to-human transmission routes, seller hand swabs address direct human exposure, and surface swabs from chicken display tables capture environment-to-human exposure risks. This triangulated sampling approach represents a novel application of One Health principles in traditional market settings in Aceh, Indonesia. Therefore, to the best of our knowledge, this is among the first studies in the region to simultaneously assess AMR in poultry, human handlers, and environmental surfaces.

### Isolation and identification of *E. coli*

The isolation of *E. coli* was conducted using the classical screening method. The collected samples were incubated at 37°C for 24 h to facilitate bacterial growth. Following incubation, the samples were serially diluted in 0.85% sodium chloride-containing sterile distilled water to maintain osmotic balance and optimize bacterial isolation.

The diluted samples were streaked onto eosin methylene blue agar (EMBA) and incubated at 37°C for 24 h under aerobic conditions. The number of *E. coli* colonies selected per sample was based on the presence of colonies exhibiting typical characteristics on EMBA medium, particularly those showing a green metallic sheen. Further confirmation was performed through Gram staining, where isolates exhibiting Gram-negative, rod-shaped morphology were considered putative *E. coli*. These presumptive *E. coli* colonies were then subjected to a series of biochemical tests for confirmation. A total of 54 *E. coli* colonies were confirmed from 174 samples collected from three traditional markets.

This study was designed as a preliminary investigation to screen for the occurrence and resistance profile of *E. coli* across traditional markets in a One Health context. Therefore, colony-forming units (CFU) were not quantified per sample, and specific molecular confirmation methods were not included. The primary focus of this study was phenotypic identification and resistance characterization. The procedures followed the standardized guidelines of the Clinical and Laboratory Standards Institute (CLSI), and to ensure consistency and reliability, all results were interpreted based on the CLSI breakpoints [14].

### Biochemical confirmation of *E. coli*

Presumptive *E. coli* colonies were biochemically characterized using the Indole, Methyl Red, Voges-Proskauer, and Citrate (IMViC) test panel, a classic but robust differentiation system within Enterobacteriaceae. This method remains essential for reliable confirmation of fecal contamination in resource-limited settings.

The confirmation process involved IMViC tests, as well as standard biochemical assays, to differentiate *E. coli* from other members of the Enterobacteriaceae family. Each test was performed using conventional media: Tryptone broth for the Indole test, MR–VP broth for Methyl Red and Voges–Proskauer assays, and Simmons citrate agar for citrate utilization, with incubation at 37°C for 24–48 h depending on the test. Positive indole and methyl red reactions, combined with negative Voges–Proskauer and citrate results, confirmed the presence of *E. coli*.

### Antibiotic susceptibility testing (Kirby–Bauer method)

The antibiotic susceptibility of *E. coli* isolates was determined using the Kirby–Bauer disk diffusion method, following the guidelines of the CLSI (2021; document M100, 31^st^ edition) [14].

Confirmed *E. coli* isolates were cultured in tryptic soy broth and incubated at 37°C for 4–6 h until the turbidity matched 0.5 McFarland standard (~1.5 × 10^8^ CFU/mL). The standardized suspensions were evenly spread onto Mueller–Hinton agar (Oxoid, UK), and antibiotic discs (Oxoid, UK) were applied using sterile forceps.

The antibiotic susceptibility test was conducted using 11 antibiotic disks, comprising tetracycline (TE) (30 μg), chloramphenicol (C) (30 μg), ampicillin (AMP) (10 μg), cephalothin (KF) (30 μg), streptomycin (S) (10 μg), gentamicin (CN) (10 μg), sulfamethoxazole/trimethoprim (SXT) (25 μg), nalidixic acid (NA) (30 μg), ciprofloxacin (CIP) (5 μg), erythromycin (E) (15 μg), and kanamycin (K) (30 μg). The Petri dishes were incubated at 37°C for 18–24 h under aerobic conditions.

The antibiotic panel used in this study was selected based on the frequency of use of these antibiotics in poultry farming in the study region and their clinical relevance in treating *E. coli* and other enteric bacterial infections. Antibiotics such as TE and S are commonly used in veterinary practices, particularly in poultry production. Drugs such as CIP, CN, and SXT are widely used in human healthcare settings. Including both veterinary and clinically significant antibiotics allows for a comprehensive assessment of AMR that reflects both agricultural practices and potential public health risks.

Following incubation, the diameters of inhibition zones surrounding the antibiotic disks were measured in millimeters (mm) using a digital caliper. The results were interpreted as susceptible (S), intermediate (I), or resistant (R) according to the CLSI 2021 breakpoint tables [14]. For the purpose of MDR classification, intermediate results were grouped together with resistant isolates. MDR was defined as resistance to at least one agent in three or more antibiotic classes, in accordance with international standards.

No automated systems were used in this study. Each test was performed in triplicate, and resistance percentages were descriptively analyzed. This standardized approach enables a comprehensive assessment of antibiotic resistance patterns in *E. coli* isolates from poultry markets, contributing to a better understanding of AMR in the poultry supply chain.

### Statistical analysis

The data obtained from this study were analyzed descriptively. The results of the microbiological and antibiotic susceptibility tests were entered into a table in Microsoft Excel for quantitative analysis. The prevalence rates of *E. coli* isolation were calculated as the number of positive samples divided by the total number of samples examined and expressed as percentages. Similarly, resistance percentages for each antibiotic were calculated as the proportion of resistant isolates out of the total number of tested isolates.

In this preliminary study, inferential statistical analyses were not performed. Therefore, the results were presented in a purely descriptive manner to provide an overview of the contamination levels and resistance patterns in the study area.

## RESULTS

### Isolation and morphological characterization of *E. coli*

Isolation of *E. coli* colonies was successfully carried out from various sample sources using selective culture media. Colonies suspected to be *E. coli* appeared with characteristic morphology, including round shape, smooth surface, and typical coloration on the selective agar. Gram staining and biochemical tests were performed to confirm the identification.

**Figure 2:**
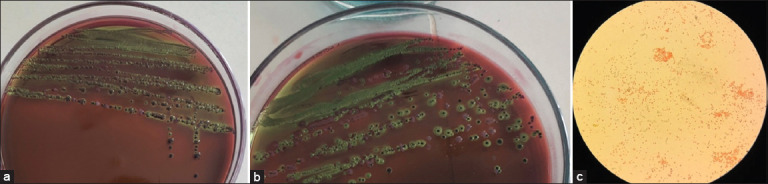
Morphological characteristics of *Escherichia coli* colonies: (a) Macroscopic appearance of isolates from fecal samples, (b) macroscopic appearance of isolates from poultry sellers’ hands on eosin methylene blue agar medium, and (c) microscopic morphology of Gram staining results of *E. coli* isolates observed with 1000× magnification.

The successful isolation of *E. coli* colonies from two different sample sources is shown in Figure 2. Colonies isolated from fecal samples exhibited a typical metallic green sheen (Figure 2a), a characteristic appearance of *E. coli* on EMBA medium, indicating lactose fermentation. Similar greenish colonies were observed from samples collected from the hands of poultry sellers (Figure 2b).

### Confirmation of *E. coli*

The *E. coli* isolates were confirmed further through Gram staining (Figure 2c) and biochemical tests. Microscopic observations showed pink-colored, rod-shaped bacteria, confirming the Gram-negative characteristics typical of *E. coli*. The uniform morphology and staining pattern of the colonies further supported the preliminary identification.

The biochemical tests also validated the metabolic characteristics of the isolates. Positive indole reactions were observed, indicated by the formation of a red ring after reagent addition. Lactose fermentation was confirmed by a purple color change in the medium, demonstrating the ability of the isolates to ferment lactose. These consistent positive reactions strongly validated the identification of *E. coli* as the bacterial isolates.

### Prevalence of *E. coli* in traditional markets

This study identified the prevalence of *E. coli* contamination in fecal and chicken meat swab samples, poultry sellers’ hands, and chicken display tables collected from one traditional market in Aceh Besar District (Lambaro) and two markets in Banda Aceh City (Al-Mahirah and Seutui). Table 1 shows the prevalence and distribution of *E. coli* isolated from the three market locations.

**Table 1:** Distribution and prevalence of *E. coli* in traditional markets in Aceh Besar District and Banda Aceh City.

Market location	Number of samples								Total sample	Prevalence of *E. coli* infection (%)

	Fecal		Chicken meat		Poultry seller’s hand		Chicken display table			
			
	+	−	+	−	+	−	+	−		
Lambaro	5	13	7	11	4	12	4	2	58	11.49 (20/174)
Al-Mahirah	11	7	6	12	3	13	3	3	58	13.2 (23/174)
Seutui	8	10	2	16	1	15	0	6	58	6.32 (11/174)
Total samples	24	30	15	39	8	40	7	11	174	31.03 (54/174)

*E. coli* = *Escherichia coli.*

The overall prevalence of *E. coli* isolated from these markets and the four sample sources was 31.03% (54/174). Contamination levels varied by market: Al-Mahirah had the highest prevalence (13.2%), followed by Lambaro (11.49%) and Seutui (6.32%). By sample type, contamination was highest in fecal samples (13.79%), followed by chicken meat swabs (8.62%), sellers’ hand swabs (4.60%), and chicken display tables (4.02%). Interestingly, *E. coli* contamination was absent in display table samples from the Seutui market.

### Antibiotic susceptibility of *E. coli*

**Figure 3:**
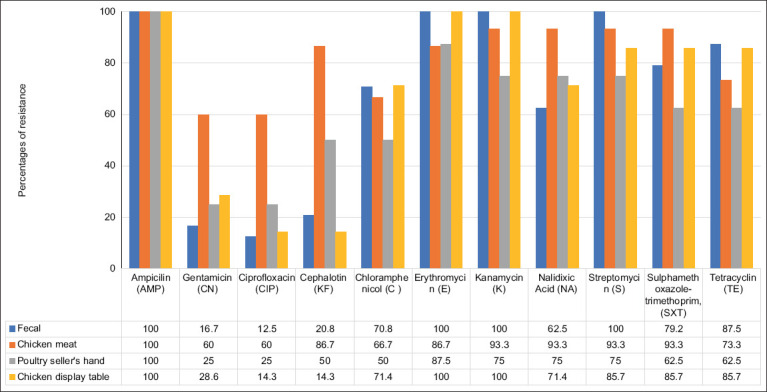
Antibiotic resistance patterns of *Escherichia coli* isolates from four different sources in three traditional poultry markets in Aceh Besar District and Banda Aceh City. All the numbers are percentages.

The susceptibility test of 54 *E. coli* isolates from four sources of samples against 11 antibiotics revealed diverse resistance patterns. All isolates collected from the three traditional markets showed resistance to the antibiotics tested. Table 2 summarizes the results. Figure 3 illustrates the antibiotic resistance patterns and MDR profiles of the isolates.

**Table 2:** Results (percentage) of antibiotic susceptibility test to isolated *E. coli* bacteria from four types of samples collected in three traditional markets in Aceh Besar District and Banda Aceh City.

Types of antibiotics used	Type of samples (susceptible, intermediate, and resistant)											

	Fecal			Chicken meat			Poultry sellers’ hand			Chicken display table		
			
	S	I	R	S	I	R	S	I	R	S	I	R
AMP	0	0	100	0	0	100	0	0	100	0	0	100
CN	66.7	16.7	16.7	20	20	60	62.5	12.5	25	71.4	0	28.6
CIP	62.5	25.0	12.5	6.7	33.3	60	62.5	12.5	25	71.4	14.3	14.3
KF	45.8	33.3	20.8	6.7	6.7	86.7	50	0	50	85.7	0	14.3
C	20.8	8.3	70.8	13.3	20	66.7	0	50	50	28.6	0	71.4
E	0	0	100	6.7	6.7	86.7	0	12.5	87.5	0	0	100
K	0	0	100	0	6.7	93.3	0	25	75	0	0	100
NA	8.3	29.2	62.5	6.7	0	93.3	12.5	12.5	75	14.3	14.3	71.4
S	0	0	100	0	6.7	93.3	0	25	75	0	14.3	85.7
SXT	4.2	16.7	79.2	6.7	0	93.3	12.5	25	62.5	14.3	0	85.7
TE	0	12.5	87.5	6.7	20	73.3	12.5	25	62.5	0	14.3	85.7

*E. coli* = *Escherichia coli*, S = Susceptible, I = Intermediate, R = Resistant, AMP = Ampicillin, CN = Gentamicin, CIP = Ciprofloxacin, KF = Cephalothin, C = Chloramphenicol, E = Erythromycin, K = Kanamycin, NA = Nalidixic acid, S = Streptomycin, SXT = Sulfamethoxazole-trimethoprim, TE = Tetracycline.

- Fecal samples: 100% resistance was observed to AMP, E, K, and S (Table 2). In addition, more than half of isolates were resistant to C (70.8%), NA (62.5%), SXT (79.2%), and TE (87.5%).
- Chicken meat swabs: 100% resistance was found only to AMP. However, more than 50% (60–93.3%) of isolates were resistant to 10 antibiotics, with the highest resistance (93.3%) against K, NA, S, and SXT.
- Πουλτρψ sellers’ hand swabs: 100% of isolates were resistant to AMP. In addition, more than 50% showed resistance to E, K, S, SXT, and TE, ranging between 62.5% and 87.5%.
- Display table swabs: 100% resistance was observed to AMP, E, and K. More than 50% of isolates were resistant to C (72.7%), NA (81.8%), S (72.7%), SXT (72.7%), and TE (81.8%).

### Variation in resistance by market and sample source

**Figure 4:**
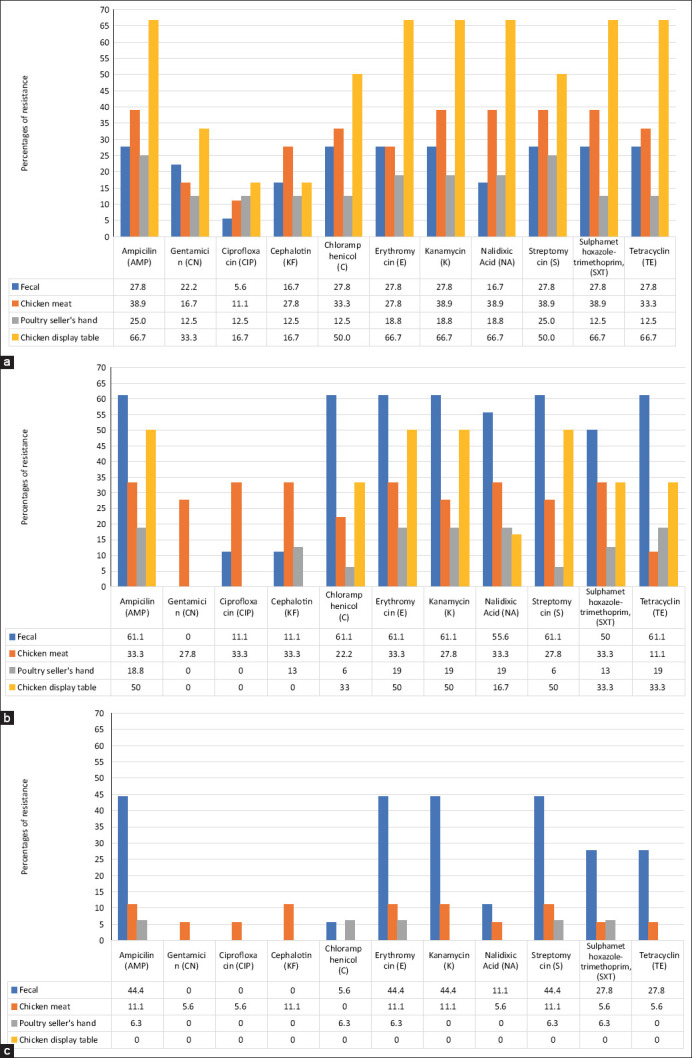
Percentage of *Escherichia coli* isolates resistant to antibiotics distributed in three traditional markets and four different sources of samples in Banda Aceh City and Aceh Besar District. (a) Lambaro market, (b) Al-Mahirah market, and (c) Seutui market. All the numbers are percentages.

Resistance patterns varied between markets and sample types (Figure 4).

- Lambaro market: Isolates from display tables showed higher resistance to 10 of 11 antibiotics compared to other sample sources, except for KF, where chicken meat isolates had higher resistance. Overall, chicken meat isolates were more resistant than fecal and seller’s hand isolates for eight antibiotics, while fecal isolates had higher resistance than hand isolates for nine antibiotics. Resistance percentages was found lowest in fecal isolates to CIP (5.6%) as compared to other sources and antibiotics.
- Al-Mahirah market: Fecal isolates displayed higher resistance to eight antibiotics compared with other sources. Display table isolates showed resistance to seven antibiotics. Interestingly, only chicken meat isolates was found resistant to CN, but none to fecal, hand, and display table isolates. Similarly, hand isolates were not resistant to CIP, while display table isolates showed no resistance to CIP, and KF.
- Seutui market: None of the display table isolates were resistant to any antibiotics. Fecal isolates, however, exhibited higher resistance to seven antibiotics compared with other sources. Seller’s hand isolates were resistant only to AMP, C, E, S, and SXT. Chicken meat isolates were found resistant to 10 of 11 antibiotics, and uniquely showed resistance to CN, CIP, and KF – all drugs of critical importance in human medicine – whereas no resistance to C was observed in chicken meat isolates.

## DISCUSSION

To the best of our knowledge, this is among the first studies in Indonesia to apply a triangulated One Health framework in traditional market settings. Previous AMR investigations in Southeast Asia [3, 4] and Indonesia [6, 7, 9] have largely relied on conventional approaches, focusing on either poultry farms or retail meat. In contrast, we employed an integrated triangulated sampling strategy by simultaneously assessing *E. coli* from animals (fecal samples and chicken meat), humans (sellers’ hands), and the environment (display tables). This design enables the identification of multiple transmission nodes within the same market ecosystem. Importantly, it also allowed us to capture post-farmgate contamination risks, which are an aspect often overlooked, and thereby offers a broader perspective on how AMR circulates and persists within informal market systems [15].

### Patterns of *E. coli* contamination

The findings of our study showed that most *E. coli* contamination was found in fecal samples, followed by chicken meat, chicken display table, and poultry sellers’ hands. This is consistent with many studies that suggested that fecal contamination serves as the primary source of *E. coli* in poultry systems and other livestock animals, which can subsequently spread to meat during slaughtering, evisceration, and processing [6, 15, 16]. Comparable findings were reported by several studies in Indonesia and Algeria, where cloacal swab samples collected from broilers also showed higher contamination rates at 76.47%–80.5%, as compared to meat samples (44.26%) [7, 11, 17].

The lower rates of meat compared with fecal samples in our study might suggest that it is the result of partial transfer that might happen during handling and processing chicken. On the other hand, the role of hygiene practices in moderating contamination is also highlighted [18]. Our study showed relatively similar contamination rates of *E. coli* observed in sellers’ hand swabs (4.60%) and display tables (4.02%). A recent study in Ghana partially followed a similar pattern, which found a lower prevalence in sellers’ hand swabs (10%) compared to raw chicken meat (20%) [17].

These results demonstrate that food handlers’ hands frequently act as secondary contamination points, resulting in a generally lower prevalence. The findings of our study, in turn, explain the pathway by which bacteria spread from chickens, to sellers, consumers, and the environment. In contrast to our findings, Adzitey *et al*. [17] reported a higher percentage of *E. coli* contamination in table swabs (30%) compared to raw chicken meat. These differences likely result from inadequate cleaning practices by poultry sellers that caused cross-contamination during handling [18]. Overall, the findings of our study support the biological fact that as long as a hygienic standard is maintained, bacteria cannot survive in an open surface environment.

### AMR burden

This study focused on market-based One Health surveillance and demonstrated a heavy resistance burden in commensal *E. coli* along the wet-market value chain. Interestingly, the results showed uniformly high resistance to AMP (100% across all sources). In general, an elevated resistance (>50%–100%) to TE, SXT, NA, S, E, and K, and lower (but non-negligible) resistance was observed to CN and CIP.

In addition, the study was able to provide a variation of resistance profiles by market and sample source (fecal, meat, sellers’ hands, display tables). These findings are similar to reports from Indonesia and the wider region that link the frequent use of older, inexpensive antimicrobials (e.g., TEs, sulfonamides, penicillins, and first-generation cephalosporins) in poultry production to high resistance in commensal Enterobacteriaceae [3, 5, 19]. At the farm level in West Java, commensal *E. coli* from broilers showed widespread MDR that might indicate on-farm antimicrobial usage, underlining the AMU–AMR selection pathway [9]. The results of our study extend this chain by showing that the occurrence resistance signatures persist from production through handling to point-of-sale surfaces and hands, critical nodes for cross-contamination.

### Resistance to key antibiotic classes

The finding of high AMP resistance (100%) is consistent with Indonesian market studies, which report dominant β-lactam resistance among poultry-associated *E. coli* [7, 10, 11]. Similarly, previous studies by Prahesti *et al*. [5] observed consistently high TE and sulfonamide resistance in Indonesian broiler systems as well as in Bangladesh [20], Sri Lanka [21], and Malaysia [4]. Therefore, the ubiquity of resistance to classes used extensively for growth promotion or metaphylaxis in Southeast Asian poultry is expressed [3, 19].

Notably, resistance to E, which is an agent with limited clinical relevance for Enterobacterales but widely used in poultry, was also high, paralleling environmental and farm studies that track selection pressure in non-enteric targets yet capture co-selection within *E. coli* populations [2, 22].

The fluoroquinolone and aminoglycoside findings in this study justify the special attention given by the World Health Organization (WHO) under “critically important” status [23]. Interestingly, the results of the measurement in this study on CIP resistance were lower than those for older classes in some sources, remained substantial in meat, and varied across markets. Notably, NA, a quinolone class indicator, showed high resistance. KF (first-generation cephalosporin) resistance was heterogeneous by market and source, which parallels the findings of previous reports on Indonesian retail meats [7, 11].

Together, this pattern suggests an ongoing selection for quinolone/fluoroquinolone resistance determinants in the poultry chain [4, 20, 21]. Importantly, the detection of resistance to CIP, a critical frontline drug in human medicine, signals a direct threat to the future of effective treatment options for foodborne infections.

From a market hygiene perspective, the presence of resistant *E. coli* on the hands and display tables of sellers indicates novel transmission vectors that extend beyond contaminated meat. It reflects lapses in sanitation during traditional or informal trading practices. In fact, traditional markets play a central role in food access in Aceh. They operate within socioeconomic settings of limited biosecurity and might represent overlooked AMR hotspots where resistant bacteria can spread rapidly among consumers and communities [17]. This interpretation underscores both the novelty and urgency of our study in framing AMR as a broader public health and social challenge.

### Market-specific variability

In this study, the findings of isolates resistant to CN were comparatively low in several markets. In general, it aligns with previous reports in Indonesia [24, 25]. It is also consistent with the observations of prior retail studies by Adzitey *et al*. [17] and Ranasinghe *et al*. [21] and the Uganda farm study, where CN use was less frequent than that of TEs or sulfonamides [26]. Based on the results, the presence of CN resistance in certain sources may occur through sporadic use, co-selection, or environmental acquisition.

The high occurrence of MDR in this study contributes to additional evidence from Indonesian value chains. Among them, MDR and extended-spectrum beta-lactamases-producing *E. coli* have been documented in broiler and market samples in West Java [7], and large MDR profiles in broiler meats were similarly noted in East Java [10, 11]. Similar results have been reported in other countries, including India [27], Sri Lanka [21], Bangladesh [20], Malaysia [4], Algeria [16], and Uganda [26]. Although species differences apply, *Salmonella* isolates in Cambodia’s farm-level data also demonstrate high resistance pressures [28].

Our study further revealed the heterogeneity of resistance distributions between markets and sources.

- Lambaro market: Isolates from chicken display tables exhibited higher resistance to 10 of 11 antibiotics than isolates from other sources, except for KF, which was more resistant in chicken meat isolates. These findings are consistent with observations from Bogor traditional markets [25], and in Burkina Faso, Africa, and underscore the role of contaminated surfaces and prolonged carcass exposure in amplifying resistance [18, 29].
- Al-Mahirah market: Fecal isolates exhibited higher resistance to eight antibiotics, followed by display table isolates. However, no resistance to CN was observed in fecal, hand, or table samples, aligning with reports of limited CN resistance due to restricted usage [4, 26].
- Seutui market: No resistance was found among display table isolates, but fecal isolates were resistant to seven antibiotics. Chicken meat uniquely harbored resistance to CN, CIP, and KF – drugs of critical human importance [23].

Perhaps the different findings between markets in our study are due to the different behaviors of sellers in maintaining the cleanliness of their facilities. In the Seutui market, cleaning practices were implemented more effectively, and poultry sellers’ stalls were better organized and separated from other commodities, resulting in no contamination on display tables and lower contamination on sellers’ hands and chicken meat compared to the other two markets. In addition, our visual observation revealed that poultry populations in Al-Mahirah and Lambaro markets were larger than those in Seutui market.

Dione *et al*. [18] suggested that differences in hygiene practices, infrastructure, and poultry handling methods may explain bacterial contamination in informal poultry markets. Markets with poor sanitation, limited access to clean water, and higher bird density are more likely to experience contamination [18, 29]. Such differences could justify the observations from Indonesian retail settings [7, 10, 11] and regional studies [16, 28].

### Transmission pathways and hygiene gaps

The side-by-side comparison of fecal, chicken meat, poultry sellers’ hands, and display tables across multiple markets is a key contribution of this study. Two important findings were observed:

1. Resistance loads on sellers’ hands and display tables for several drug classes approximated or exceeded those in meat. This explains the hygiene gaps documented in traditional markets, such as poor utensil sanitation, reuse of water, and inadequate separation of clean or dirty zones, which facilitate cross-contamination and amplification of resistant bacteria [29]. Visual observation showed sellers frequently showered water on the table to keep it clean. However, rinsing carcasses under unhygienic market conditions does not reliably reduce bacterial contamination [18].
2. Intermarket variability suggests that local practices, such as cutting board use, blade sterilization, handwashing frequency, turnover of display surfaces, and temperature control, modulate resistance distributions.

Together, these patterns underscore fecal contamination as the primary entry point for *E. coli*, with meat, hands, and display tables serving as mediators for onward spread in market environments, consistent with global retail studies [17, 21, 27].

### One Health and global context

The concurrent detection of resistant *E. coli* in fecal samples (a proxy for on-farm selection), chicken meat (a food pathway), hands (a human interface), and surfaces (an environmental component) illustrates the interconnected compartments emphasized in One Health AMR frameworks [2, 30].

The findings of this study complement evidence from Indonesia, which links higher antimicrobial inputs to higher MDR prevalence [9], and align with reports from integrated agroforestry–livestock settings [6]. Together with Southeast Asian antimicrobial use characterizations showing easy access to drugs and limited stewardship [3, 19], our data support interventions that span:

1.Farm stewardship – Restricting non-therapeutic use, veterinary oversight, and alternatives such as vaccination and biosecurity.
2.Hygiene upgrades – Improvements in slaughter and market infrastructure (running water, surface sanitation, cold chain).
3.Integrated surveillance – Including environmental and human-contact nodes at markets [2, 23, 30].

The results also align with global perspectives. MDR *E. coli* has been documented in Indian retail chicken meat [27], as well as in Ugandan and Burkinabe markets [18, 26, 29], and in Southeast Asian poultry systems [3, 19]. Therefore, this study contributes to the evidence positioning Southeast Asian traditional markets as critical nodes in the global AMR web, where food safety, public health, and trade intersect.

### Limitations and future research

Despite the consistency of findings and the use of standard microbiological methods, this study has limitations. The lack of molecular confirmation of resistance genes or plasmid characterization restricts insight into the genetic determinants of MDR. We also did not perform quantitative risk factor analyses (e.g., sellers’ hygiene practices, infrastructure, antibiotic use patterns), which could have explained observed contamination.

Future research should incorporate molecular approaches such as polymerase chain reaction detection of resistance genes and whole-genome sequencing to identify plasmid-mediated resistance. Longitudinal surveillance across seasons and market cycles is also needed. Importantly, integrating traditional market surveillance into Indonesia’s national AMR frameworks would enhance policy relevance, ensuring local data inform antimicrobial stewardship, food safety regulation, and health action plans.

## CONCLUSION

This study, to our knowledge, is the first to employ a triangulated One Health framework in Indonesian traditional markets and revealed significant contamination and AMR burdens in commensal *E. coli*. Overall prevalence reached 31.03%, with the highest contamination found in fecal samples (13.79%), followed by chicken meat (8.62%), sellers’ hands (4.60%), and display tables (4.02%). Resistance patterns were alarming, with 100% of isolates resistant to AMP across all sources, and consistently high resistance (>50%) to TE, SXT, NA, S, E, and K. Although lower, resistance to CIP and CN, classified by the WHO as critically important for human medicine, was also detected. Market-level variability further demonstrated that differences in hygiene practices and infrastructure contribute to heterogeneity in contamination and resistance profiles.

The practical implications of these findings are substantial. Traditional markets remain central to the poultry trade and food access in Aceh, but they also serve as overlooked hotspots for AMR dissemination, where resistant bacteria can move from chickens to meat, from sellers to surfaces, and ultimately to consumers. These results underscore the urgent need for improved hygiene infrastructure (e.g., clean water, cold chain, surface sanitation), seller training in biosecurity, and stricter regulation of antimicrobial use in poultry farming. Furthermore, routine surveillance should expand beyond farms and retail meat to include human and environmental nodes within market ecosystems.

The strength of this study lies in its triangulated One Health sampling design, which allowed the simultaneous investigation of animal, human, and environmental contamination pathways. This holistic approach provided a more complete understanding of AMR dynamics in traditional markets than farm- or meat-focused studies alone.

In conclusion, the persistence of MDR *E. coli* in Aceh’s traditional markets highlights both the local and global relevance of post-farmgate surveillance. Addressing AMR in such informal food systems requires coordinated interventions at multiple levels, including prudent antimicrobial stewardship, hygiene upgrades, and integration of markets into national AMR action plans. By documenting the resistance burden at the animal–human–environment interface, this study provides context-specific evidence that supports Indonesia’s contribution to global One Health strategies for controlling AMR.

## AUTHORS’ CONTRIBUTIONS

TRF: Conceived and designed the study, developed the methodology, and drafted the original manuscript. MA and WES: Data collection, laboratory analyses, investigation; drafted sections of the manuscript and critically revised the text for important intellectual content. FJ, AA, and II: Data validation, data analysis, and conducted literature searches. MS, EE, and SRA: Data interpretation, visualization, and critically revised the manuscript. ML: Supervision and critical review of the manuscript. All authors have read and approved the final version of the manuscript.
